# Adulteration Detection of Pork in Mutton Using Smart Phone with the CBAM-Invert-ResNet and Multiple Parts Feature Fusion

**DOI:** 10.3390/foods12193594

**Published:** 2023-09-27

**Authors:** Zongxiu Bai, Rongguang Zhu, Dongyu He, Shichang Wang, Zhongtao Huang

**Affiliations:** 1 College of Mechanical and Electrical Engineering, Shihezi University, Shihezi 832003, China; baizx1029@163.com (Z.B.); hedy_1221@163.com (D.H.); scw_shzu@163.com (S.W.); hzt_1120@163.com (Z.H.); 2 Key Laboratory of Northwest Agricultural Equipment, Ministry of Agriculture and Rural Affairs, Shihezi University, Shihezi 832003, China; 3 Engineering Research Center for Production Mechanization of Oasis Characteristic Cash Crop, Ministry of Education, Shihezi University, Shihezi 832003, China

**Keywords:** adulterated mutton, quantitative detection, smart phone, deep learning, CBAM-Invert-ResNet

## Abstract

To achieve accurate detection the content of multiple parts pork adulterated in mutton under the effect of mutton flavor essence and colorant by RGB images, the improved CBAM-Invert-ResNet50 network based on the attention mechanism and the inversion residual was used to detect the content of pork from the back, front leg, and hind leg in adulterated mutton. The deep features of different parts extracted by the CBAM-Invert-ResNet50 were fused by feature, stitched, and combined with transfer learning, and the content of pork from mixed parts in adulterated mutton was detected. The results showed that the *R*^2^ of the CBAM-Invert-ResNet50 for the back, front leg, and hind leg datasets were 0.9373, 0.8876, and 0.9055, respectively, and the RMSE values were 0.0268 g·g^−1^, 0.0378 g·g^−1^, and 0.0316 g·g^−1^, respectively. The *R*^2^ and RMSE of the mixed dataset were 0.9264 and 0.0290 g·g^−1^, respectively. When the features of different parts were fused, the *R*^2^ and RMSE of the CBAM-Invert-ResNet50 for the mixed dataset were 0.9589 and 0.0220 g·g^−1^, respectively. Compared with the model built before feature fusion, the *R*^2^ of the mixed dataset increased by 0.0325, and the RMSE decreased by 0.0070 g·g^−1^. The above results indicated that the CBAM-Invert-ResNet50 model could effectively detect the content of pork from different parts in adulterated mutton as additives. Feature fusion combined with transfer learning can effectively improve the detection accuracy for the content of mixed parts of pork in adulterated mutton. The results of this study can provide technical support and a basis for maintaining the mutton market order and protecting mutton food safety supervision.

## 1. Introduction

Mutton is popular because of its rich protein content, low cholesterol and fat content, unique flavor, and delicate taste [1]. The prices of mutton have been rising in recent years. Under the temptation of huge economic benefits, some illegal traders take the risk of mixing low-value meat, such as pork, with mutton for the sale of adulterated products [2]. At the same time, illegal traders add food additives, such as mutton flavor essence and colorant, to the adulterated mutton to further achieve the effect of “fake with real”. It not only seriously infringes the economic interests of consumers and destroys the market order but also poses a threat to the health of consumers and causes food safety problems. Therefore, it is urgent to seek a rapid and accurate method for the detection of adulterated pork in mutton under the action of mutton flavor essence and colorant.

At present, the detection methods for meat or food adulteration mainly include sensory tests, chromatographic analysis [3], immunoassay [4], DNA analysis [5], intelligent sensing technology [6,7], optical-colorimetric methods [8], and modern optical rapid detection technology. With the increasing level of adulteration, sensory analysis has been completely unable to meet the demand of current detection. The methods of chromatography, immunoassay, and DNA analysis require expensive instruments and complex pretreatment methods, so it is becoming increasingly difficult to meet the requirements of rapid and accurate detection. With the development of artificial intelligence, modern optical rapid detection methods have developed rapidly. Among them, with the popularity of smartphones and the great improvement in computing power, the development of mobile phone camera technology has made rapid progress. Smartphones have the characteristics of convenience, speed, and high calculation accuracy, and they have been widely used in the field of food detection [9,10]. In recent years, there have also been some studies on the use of smartphone image technology to classify meat parts [11], detect adulteration [12,13], and perform other functions. Previous studies have shown that there are certain differences in different parts of meat. The images taken with a smartphone can be used to detect mutton parts and meat adulteration. However, there are few studies on detecting meat adulteration (using different and mixed parts) with smartphone images. In addition, the detection of adulterated pork content in mutton using RGB images of smartphones under the effect of mutton flavor essence and colorant presents some challenges.

With the development of intelligence and information technology, deep learning has played an irreplaceable role in the fields of artificial intelligence, such as computer vision and natural language processing. As a typical representative of deep learning, a convolutional neural network (CNN) can effectively learn feature expressions from a large number of sample images and enhance the generalization ability of the model. It has the advantages of fast and accurate image processing and is currently widely used in the detection of agricultural products [14,15,16]. With the continuous expansion of computing requirements, the network layers of CNN models were continuously deepened to improve network performance. As a result, the model began to have problems such as gradient disappearance and network degradation. He et al. [17] proposed the ResNet network that used residual structure in the model to effectively solve the above problems. The superior performance has enabled it to achieve good results in many tasks, such as image classification [1,18,19], object detection [20,21], and so on. However, the ResNet network still has problems such as too many network parameters and slow convergence speed. Studies have shown that the inverted residual structure in the MobileNet can improve the convergence speed and reduce the model parameters by reducing the computation amount of high-dimensional space and the memory requirement so as to realize the lightweight of the model structure [22,23]. Cui et al. [24] added the inversion residual structure in the MobileNet v2 network to the DenseNet network model and proposed an improved lightweight DenseNet network to effectively realize the surface defect detection of mobile phone screens. Xu et al. [25] introduced an inverted residual structure into YOLOv5 for gesture recognition, and the model size was reduced by 33 MB compared with that before the improvement. Although the inverted residual structure meets the requirements of a lightweight model, its ability to learn features with small differences is limited. There is little difference in the characteristics of adulterated mutton with different contents of pork under the influence of additives such as mutton flavor essence and coloring agent, and it is still difficult to accurately predict its content by existing models [26]. The convolutional block attention module (CBAM) [27] can effectively improve the accuracy of the model by using the spatial and channel features of the images to redistribute the feature weights and strengthen the feature differences of the image. Du et al. [28] effectively classified the quality of plug seedlings using the improved CNN based on the attention mechanism. Zhang et al. [29] improved the YOLOv4 model with the CBAM to realize sheep facial biometrics recognition. The results were compared with other different object detection models, and it was proved that the improved model had good recognition performance. The existing research has proved that adding the CBAM to deep learning models can effectively improve the performance of the model. At present, there is no report on the use of the CBAM to improve the ResNet50 network for the detection of the content of pork from different parts in adulterated mutton. However, most of the adulterated mutton on the market is mixed with multiple parts of pork. Previous studies have shown that there was some difference in different parts of the meat [11,19]. The detection model established by using a single part makes it difficult to detect the content of pork from mixed parts in adulterated mutton. Feature fusion can comprehensively utilize the image features of multiple parts and complement the advantages of multiple features [30]. It is helpful to establish a more accurate adulteration detection model for mixed parts. Although the models established by using fusion features realize the advantages of multiple features to a certain extent and meet the basic training needs when detecting the content of pork from mixed parts adulterated in mutton, the results of the model are often not accurate enough because of the difference between the fusion features and the actual features. Transfer learning uses the “knowledge” learned from previous tasks, such as data characteristics and model parameters, to assist the learning process in the new domain and obtain its own model [31,32]. Therefore, when the model mixed parts are established, the prior parameters of the model built by a single part are transferred by transfer learning, and the models are fine-tuned by fusion features [33]. Based on the full use of fusion features, the real features of adulterated meat in each part can be further extracted. At present, there are no reports on the improvement of the ResNet50 network using the CBAM to detect the content of pork from different parts in adulterated mutton and using feature fusion combined with transfer learning for the detection of mixed parts.

To sum up, to quickly and accurately detect the content of specific and mixed parts of pork in adulterated mutton under the effect of mutton flavor essence and colorant using RGB images of the smartphone, an improved CBAM-Invert-ResNet50 based on the attention mechanism and inversion residual structure was used. The specific work of the current study is as follows: (1) The images of minced pork of different proportions (10, 20, 30, and 40%) from three parts (back, front leg, and hind leg) in adulterated mutton under mutton flavor essence and colorant were collected by a smartphone. (2) The effect of the improved network model on the feature extraction of different amounts of pork in adulterated mutton was analyzed by feature visualization. (3) The detection model of the content of pork from different parts adulterated in mutton was established using the improved network and compared with the conventional network model. (4) The features of different parts were fused by feature stitching and combined with transfer learning to detect the content from mixed parts in adulterated mutton. The results provide strong evidence for market regulators to crack down on the adulteration of mutton. At the same time, our study also provides a certain theoretical basis and technical support for the quantitative detection of ingredient content in agricultural and livestock products using images combined with deep learning.

## 2. Materials and Methods

### 2.1. Sample Preparation

**Figure 1:**
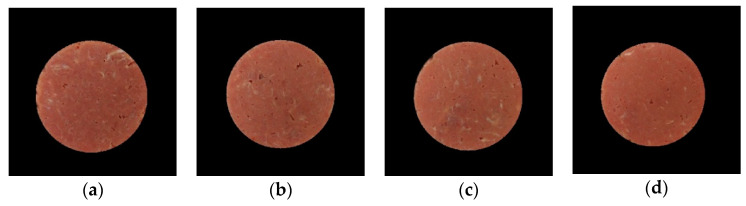
Image of different proportions of mutton adulteration with pork from the back: (**a**) 10%; (**b**) 20%; (**c**) 30%; (**d**) 40%.

Fresh mutton from the hind leg and fresh pork from different parts (back, foreleg, and hind leg) were selected to make adulterated mutton samples in this study. Mutton flavor essence and colorant were used to further interfere with the adulterated mutton samples to bring them closer to reality. The mutton flavor essence was purchased from Qingdao Xianghaisheng Food Ingredients Co., Ltd. (Qingdao, China), and the Monascus red colorant was purchased from Guangdong kelong biotechn.co., Ltd. (Jiangmen, China). Fresh hind leg meat of mutton and different parts of pork were purchased from the Youhao supermarket of Shihezi City in Xinjiang, and all of them met the quarantine standards. The meat was transported to the laboratory in an incubator. Adulterated mutton samples were prepared according to the following procedure. First, the obvious fascia and tissue on the surface of the meat were removed, and the meat was ground into 3 to 5 mm minced meat particles. After being marked and sealed with plastic wrap, the meat was stored in a refrigerator at −5 °C for subsequent use. The solvent of mutton flavor essence and colorant was obtained according to the food safety code. The mutton flavor essence solvent with a mass concentration of 0.05 g/mL was obtained by dissolving mutton flavor essence in distilled water at a dosage of 3 g per kilogram of pork and stirring for 5 min. The 0.001 g/mL solvent of the Monascus red colorant was obtained by dissolving the Monascus red colorant in distilled water at a dosage of 0.5 g per kilogram of pork. Then, the two solvents were mixed at a ratio of 1:1 and stirred for 10 min. The minced pork from different parts was soaked in the mixed solvent for 20 min, and the residual liquid on the surface was removed after the solvent was fully immersed in the minced pork. Finally, different parts of minced pork mixed with mutton flavor essence and colorant were mixed into minced mutton at different ratios (10, 20, 30, and 40%) to make adulterated mutton samples. Each sample was obtained from about 30 g of fully mixed minced meat, which was placed in a petri dish with a diameter of 6 cm and compacted to obtain a smooth surface. Eight samples were prepared from each part and each ratio of pork adulterated mutton. A total of 96 (8 × 4 × 3 = 96) samples were prepared from three parts with four ratios per part. The prepared samples were stored in a refrigerator at −5 °C for image data acquisition. The prepared various samples are shown in Figure 1.

### 2.2. Sample Image Acquisition and Pretreatment

#### 2.2.1. Sample Image Acquisition

**Figure 2:**
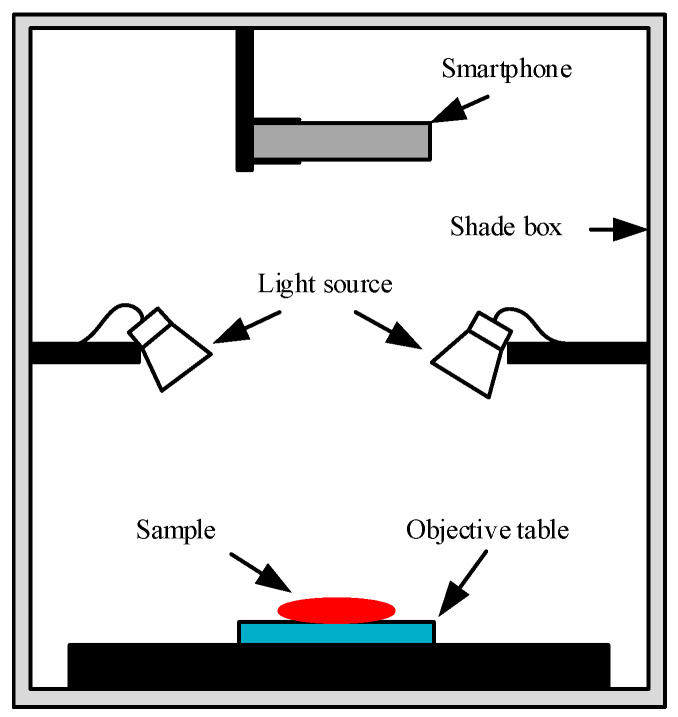
Schematic diagram of mobile phone image data acquisition system.

The mobile phone used for sample image acquisition was Huawei P40, and the camera model was ANA-AN00. Images were acquired with a camera sensitivity of 500, aperture of f/1.9, exposure time of 1/100, focal length of 7 mm, color temperature parameter of 4500 K, image resolution of 6144 × 8192 pixels, and image acquisition height of 18 cm. The ambient temperature of the laboratory was 26 ± 1 °C, and the relative humidity was 30 ± 5%. A schematic diagram of the sample image acquisition device is shown in Figure 2. There was a constant light source on the top of the dark box, and the mobile phone was fixed with a tripod. After adjusting the acquisition height of the mobile phone and the camera parameters, the images were collected. One image was collected for each sample, making it a total of 96 images.

#### 2.2.2. Image Preprocessing

**Figure 3:**
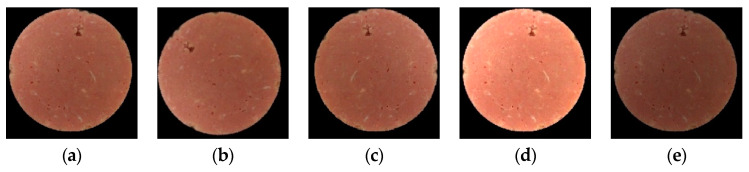
Preprocessed images: (**a**) original image; (**b**) random rotation; (**c**) mirror; (**d**) increased brightness; (**e**) decreased brightness.

In order to reduce the interference factors of the image background, the HoughCircles detection algorithm was used to extract the region of interest (ROI) of samples. In the training of deep learning models, the effect is often not ideal when the amount of data is small. To learn enough features, deep learning models need to input a large amount of data. The sample images were expanded by randomly rotating and mirroring the original images in this study. The process of random rotation was as follows: The rotation threshold was set to 0.3, and two random seeds generated random numbers between 0 and 1. When the random number generated by the No. 1 random seed was greater than 0.3, the image was rotated at the center of the origin by 360° times the random number generated by the No. 2 random seed. In addition, the brightness of the image was randomly increased and decreased to exclude the influence of different illumination intensities on the image. The process was similar to the random rotation. The preprocessed images are shown in Figure 3.

### 2.3. Production of Datasets

#### 2.3.1. Datasets of Pork from Different Parts in Adulterated Mutton

The data were divided into three datasets according to the part of pork adulterated in mutton: the back, front leg, and hind leg. Each dataset contained four adulteration ratios: 10%, 20%, 30%, and 40%. First, 1/3 of the images were taken from each dataset as an independent validation set. A total of 700 images for the independent validation set were obtained by data augmentation. Then, the remaining 2/3 data of each set were divided into a training set and a test set according to the 3:1 ratio. All images were expanded according to the methods in Section 2.2.2. The images from each dataset were expanded to obtain 1575 images for the training set and 525 images for the test set.

#### 2.3.2. Datasets of Pork from Mixed Parts in Adulterated Mutton

The datasets of pork from mixed parts in adulterated mutton contain all data from three parts. First, 1/3 of the data were taken from each part dataset as an independent validation set. A total of 2100 images for the independent validation set were obtained by data expansion. Then, the remaining 2/3 data were divided into the training set and test set according to 3:1. A total of 4725 images for the training set and 1575 images for the test set were obtained.

Due to the large size of the extended image, it takes a long time to train the model. To reduce the computational load and operation time of CNN, the expanded images of all datasets were compressed to 224 × 224 pixels.

### 2.4. Construction of the Model

#### 2.4.1. Construction of the CBAM-Invert-ResNet50 Model

The ResNet network effectively solves the problems of gradient disappearance and network degradation in the deep CNN model by using a residual structure. However, the ResNet network still has problems such as too many network parameters and slow convergence speed, which is not conducive to porting to mobile terminals. Referring to the lightweight idea of the MobileNet, the inverted residual structure was used to replace the original residual structure in the ResNet50 network in this study, which could improve the convergence speed of the model and reduce the model parameters. Existing studies have shown that attention mechanisms can make full use of the spatial and channel features of the images [27,34]. It strengthens the feature differences of the images and effectively improves the accuracy of the model through the adaptive allocation of feature weights. With the effects of additives such as mutton flavor essence and colorant, the characteristics of adulterated mutton meat with different pork content show little difference [26]. Therefore, the feature differences between adulterated mutton with different pork content can be strengthened by adding a CBAM attention mechanism to the ResNet50 network. By strengthening the weight allocation of important features, the detection efficiency of the model for adulteration content was improved. Based on the ResNet50 network combined with the CBAM attention mechanism, our research team proposed a lightweight inversion residual network CBAM-Invert-ResNet50 [35]. It was used to classify and detect mutton, adulterated mutton, and pork. However, its feasibility in quantitative detection needs to be further verified. Therefore, this study aimed to explore the feasibility of using the CBAM-Invert-ResNet50 to detect the content of pork from different parts in adulterated mutton and combine feature fusion and transfer learning to achieve an accurate prediction of adulteration content in mixed parts.

**Figure 4:**
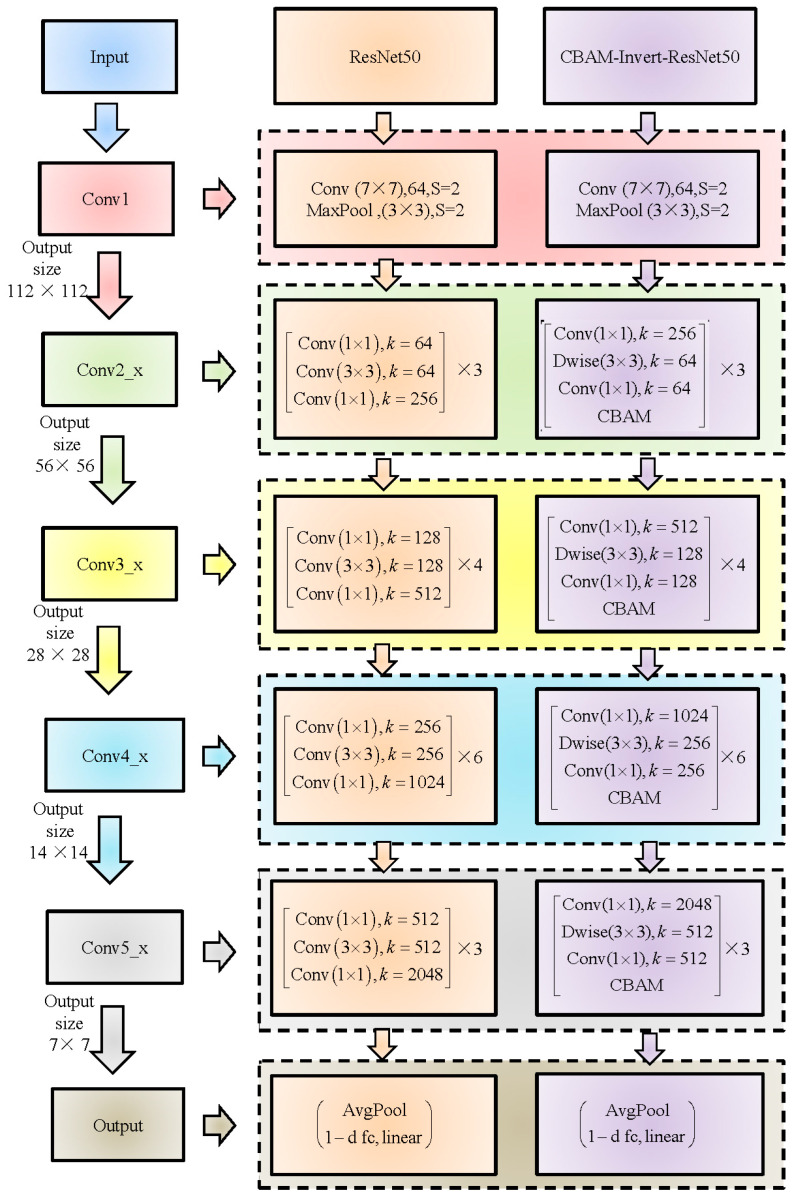
Comparison of network structure between the CBAM-Invert-ResNet50 and ResNet50.

The CBAM-Invert-ResNet network is mainly composed of seven parts: convolutional layer, pooling layer, normalization layer, inverted residual structure, CBAM structure, and fully connected layer. The structure of the CBAM-Invert-ResNet50 and ResNet50 is shown in Figure 4. The CBAM-Invert-Resnet50 is obtained by replacing the residual structure in the ResNet50 network with the inverted residual structure and adding the CBAM module after each inverted residual structure.

#### 2.4.2. Feature Fusion

**Figure 5:**
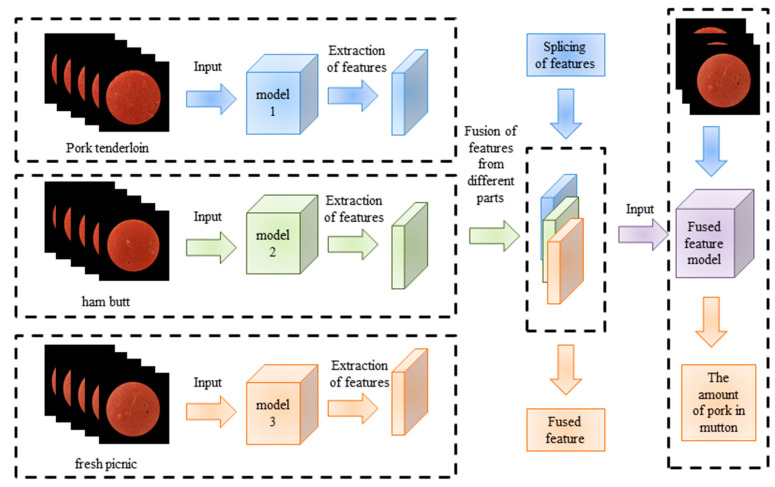
The diagram of feature fusion.

In order to realize the adulterated mutton with multiple pork parts, the feature fusion method was used to stitch the features of different parts and construct the model for the detection of the content of pork from mixed parts in adulterated mutton. The feature fusion method can comprehensively utilize a variety of image features and complement the advantages of multiple features to improve the accuracy and robustness of the model. According to the sequence of fusion and prediction, feature fusion was divided into early fusion and late fusion. Early fusion is first achieved by fusing the features of multiple network layers and then by using the fused features for model training. Late fusion improves the detection performance by combining the detection results of different layers. Before the final fusion is completed, the model starts to perform detection on the partially fused layer. Multiple detection results of multiple layers will be fused. The mixed dataset contains the back, foreleg, and hind leg datasets. Therefore, a series of feature fusion in the early fusion method was selected to join the features of the three detection models of the back, front leg, and hind leg to improve the accuracy of the model. First, the back, front leg, and back leg datasets were input into models 1, 2, and 3 for training, respectively. The features of the back, front leg, and back leg datasets were extracted using models 1, 2, and 3, respectively. Then the features extracted by the three models were stitched to obtain the fusion features. Finally, the fusion features were input into the feature fusion model for training. The feature fusion process is shown in Figure 5.

#### 2.4.3. Transfer Learning

When detecting the adulteration content of pork from mixed parts in adulterated mutton, the results of the model are often not accurate because of the difference between the fusion characteristics and the actual characteristics. Therefore, it is necessary to further extract the real features on the basis of making full use of the fused features. Transfer learning combined with fine-tuning was used to achieve the detection of adulteration content in the mixed part in this study. Fine-tuning was used to obtain data features or model parameters in both the original and new domains by freezing part of the convolutional layers of the pretrained model (usually the convolutional layers close to the input because these layers retain a large amount of underlying information) and training the remaining convolutional layers and fully connecting layers again. In this study, after the fusion features were fed into the pretrained model, the fusion features of the back, front leg, and hind leg datasets and the true features of the mixed dataset could be obtained by fine-tuning. The difference between the fused features and the actual features was eliminated by this method. Based on making full use of the fused features, the model further extracted the true features of the mixed dataset to improve the accuracy and robustness of the model.

### 2.5. Test Environment and Model Evaluation

#### 2.5.1. Evaluation Criteria of the Model

When establishing the adulteration content prediction model, the predictive effect of the model was evaluated by calculating the correlation coefficient *R*^2^ and root mean square error RMSE of the model. Their calculation equations are shown in (1) and (2):

(1) $$R^{2}=\frac{{\sum \left(\hat{x}i-\bar{x}\right)}^{2}}{\sum {\left(xi-\bar{x}\right)}^{2}}$$

(2) $$\mathrm{RMSE}=\sqrt{\frac{1}{n-1}\sum {\left(\hat{x}i-xj\right)}^{2}}$$

where ${\hat{x}}_{i}$ represents the predicted value, $x_{j}$ represents the actual value, and $\overset{¯}{x}$ represents the mean value of the actual value. *R*^2^ is the correlation between the predicted value of the model and the actual value, and a larger value of *R*^2^ indicates a stronger correlation between the two. The RMSE represents the deviation between the predicted value of the model and the actual value, and a smaller value of RMSE indicates a smaller prediction error of the model.

#### 2.5.2. Performance Evaluation of the Model

A boxplot is often used to reflect the characteristics of the distribution of the original data. It can also be used to compare the distribution characteristics of multiple groups of data. In this study, a boxplot was used to visually evaluate the stability of the model. In the boxplot, the data were divided into 4 equal fractions after being arranged from large to small. The three quartiles were the first quartile (Q1), the second quartile (Q2), and the third quartile (Q3) in descending order. In the boxplot, the top and bottom edges of the box are the third quartile (Q3) and first quartile (Q1) of the data, respectively. The entire box contains 50% of the data. IQR (Inter Quartile Range) is the interquartile range, and its formula is shown in (3):

IQR = Q3 − Q1(3)

The upper and lower short horizontal lines represent the minimum and maximum data values except for outliers, respectively, and their equations are shown in (4) and (5):

Min = Q1 – 1 *×* IQR (4)

Max = Q3 + 1 *×* IQR (5)

The variation range of IQR in the boxplot represents the distribution of the predictive value of the model for the dataset. The smaller the value, the more concentrated the distribution of the predictive value of the model. It indicates that the stability of the model is better.

#### 2.5.3. Model Test Environment

The hardware used in this study included Intel^®^CoreTM i7-10750HCPU @ 2.60 GHz processor, 16 GB memory, and NVIDIA GeForce RTX 2060 graphics card. The software included the operating system Windows 10 (64-bit), the programming language Python 3.8, the deep learning framework TensorFlow 2.3.0, General-purpose computing architecture CUDA 10.1.243, and GPU acceleration library CUDNN 7.4.1.

## 3. Results and Discussion

### 3.1. Visualization and Comparison of Depth Features Extracted by Different Models

**Figure 6:**
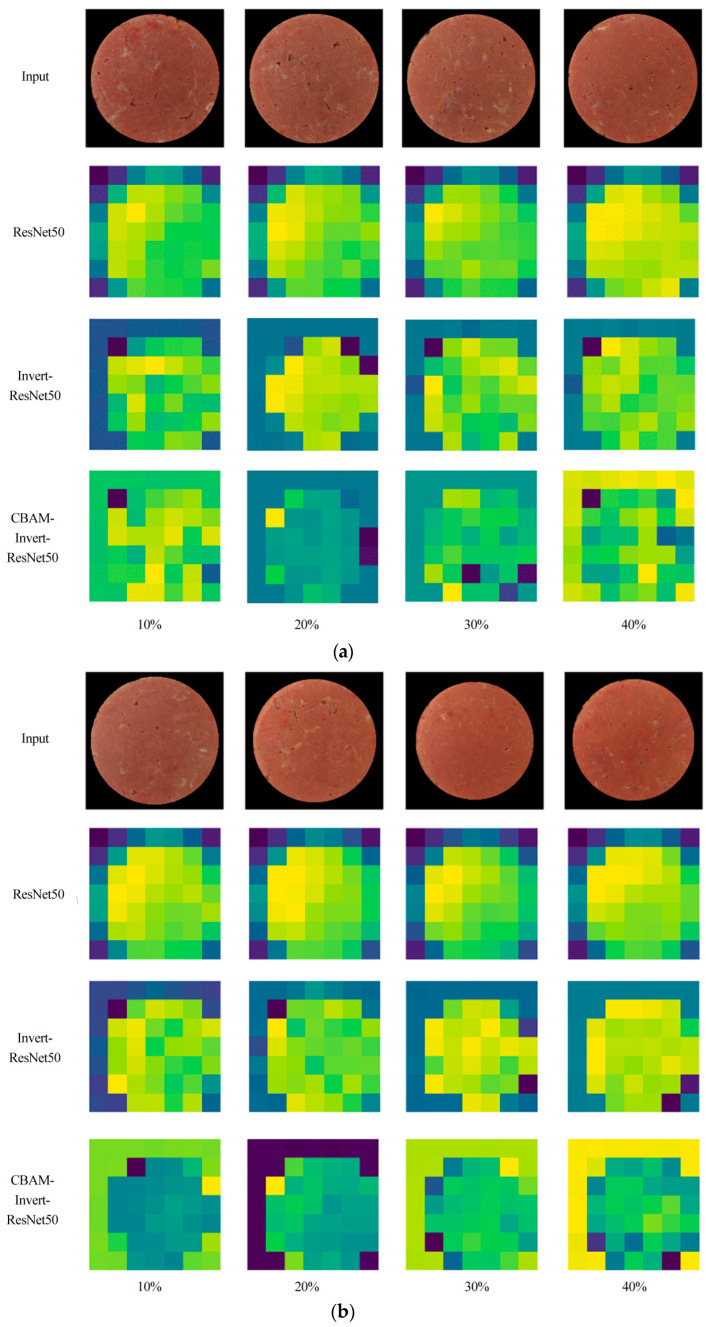

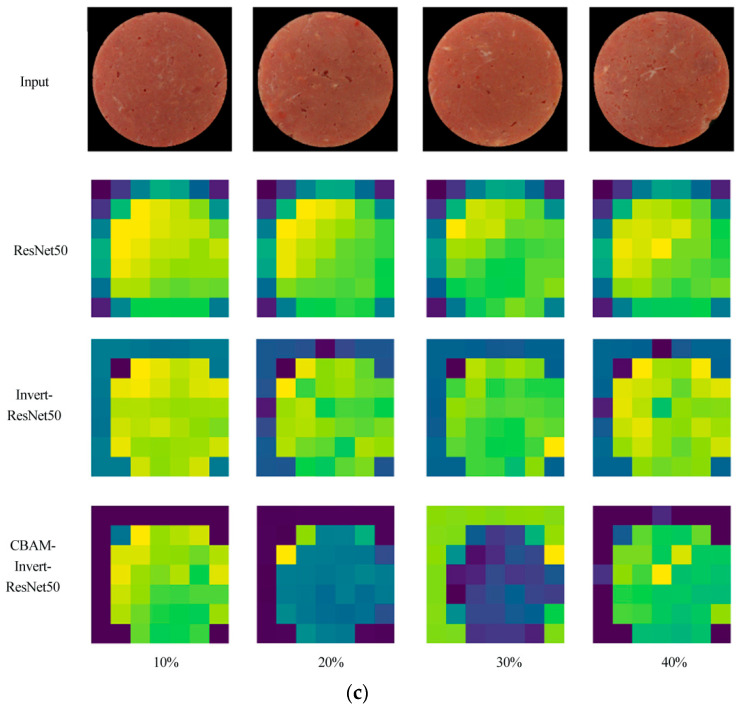
Visualization and comparison of depth features of pork from different parts (back, front leg, and hind leg) in adulterated mutton extracted by different models: (**a**) back; (**b**) front leg; (**c**) hind leg.

To explore the effect of the improved model based on the attention mechanism on the feature extraction of different pork content from different parts in adulterated mutton, the models of the ResNet50, Invert-ResNet50, and CBAM-Invert-ResNet50 were used to extract the features of the original images of samples. The output features of the last layer for the three network models are visualized, as shown in Figure 6.

In Figure 6, the columns represent the adulteration content, and from left to right are images of adulterated mutton with 10%, 20%, 30%, and 40% pork. The original input image of the sample is presented in the first row. The second, third, and fourth rows are the output features extracted by the ResNet50, Invert-ResNet50, and CBAM-Invert-ResNet50 models, respectively. It can be concluded that for the dataset of mutton adulterated with pork from the back, front leg, and hind leg, it is difficult to directly distinguish the differences in raw images of mutton adulterated with different contents of pork. After processing with the ResNet50 and Invert-ResNet50 network models, the differences in the output feature of the four proportions for adulterated mutton images are still small. Their colors and shapes are visually similar. After processing with the CBAM-Invert-ResNet50 network model, the color of the output features for the four proportions of the adulterated mutton images in the visualization map has obvious differences. The main reason is that the CABM attention mechanism can enlarge the receptive field, create dependencies between different channels, and strengthen the weight allocation of more important features [27]. The above analysis shows that the addition of the CABM in the model can strengthen the differences in the characteristics of mutton with different levels of pork adulteration, which is conducive to the rapid and accurate prediction of the content of pork from different parts in adulterated mutton under the effects of mutton flavor essence and colorant.

### 3.2. Lightweight Analysis of Improved Model

**Figure 7:**
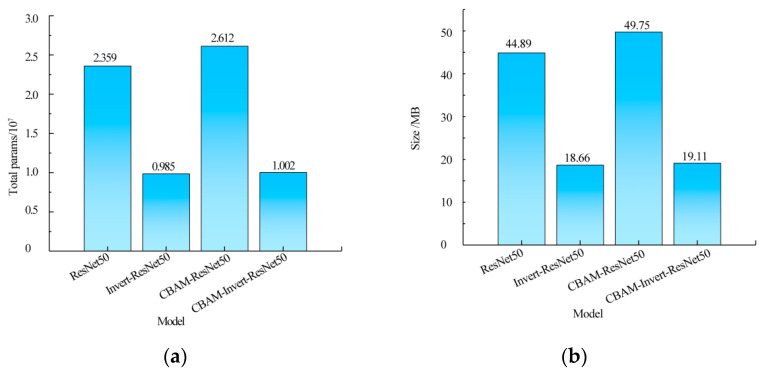
The comparison of the CBAM-Invert-ReNet50 with the ResNet50, Invert-ResNet50, and CBAM-ResNet50 in model size and parameters: (**a**) parameters count; (**b**) model size.

In order to verify the effect of the inverted residual structure on the complexity of the adulteration detection model, the model size and the number of parameters were used to measure the lightweight degree of the model. The model size and the number of parameters for the CBAM-Invert-ResNet50 model and ResNet50, Invert-ResNet50, and CBAM-ResNet50 models are shown in Figure 7.

Figure 7 shows that the model of the Invert-ResNet50 was obtained by using the inverted residual structure to replace the original residual structure in the ResNet50. Compared with ResNet50, the total number of parameters of the Invert-ResNet50 was reduced by 58.25%, from 2.359 × 10^7^ to 9.85 × 10^6^, and the size of the model was reduced from 44.89 MB to 18.66 MB, with a reduction of 58.43%. The CBAM-ResNet50 model was obtained by directly introducing the CBAM attention mechanism into the ResNet50 model. Compared with ResNet50, both the number of parameters and the model size were increased, which did not meet the requirements of the model lightweight. Therefore, the CBAM-Invert-ResNet50 network was obtained by replacing the residual structure in the CBAM-ResNet50 with the inverted residual structure. The number of parameters reduced to 2.612 × 10^7^ from 1.002 × 10^7^, with a reduction of 61.64%. The size of the model was reduced to 19.11 MB from 49.75 MB, with a reduction of 61.59%. Compared with the ResNet50 and CBAM-ResNet50, the number of parameters of the Invert-ResNet50 and CBAM-Invert-ResNet50 networks was significantly reduced, indicating that the inverse residual structure could significantly reduce the number of network parameters of the model, thus reducing the volume of the model and realizing the lightweight of the model structure. The results were consistent with those reported in previous studies. Cui et al. added the residual structure to the DenseNet network, and the number of parameters in the model was reduced from 1.08 × 10^7^ to 0.89 × 10^7^ [24]. Xu et al. added the inverted residual structure in YOLOv3 and combined it with depthwise separable convolution to recognize the gesture, and the size of the model was only 0.89 M [25]. Compared with the Invert-ResNet50, the number of parameters of the CBAM-Invert-ResNet50 increased by only 1.73%, and the model size increased by 2.41%. However, the attention mechanism could strengthen the features of different pork content in pork-adulterated mutton. It made the model easier to realize the rapid and accurate prediction of the content of pork in adulterated mutton under the action of mutton flavor essence and colorant. Therefore, the CBAM-Invert-ResNet50 network could not only meet the lightweight requirements of the model but also ensure the precision of the model.

### 3.3. The Content Detection Model of Adulterated Mutton with Pork from Different Parts

#### 3.3.1. Results of the CBAM-InvertResNet50 Model

To verify the feasibility of the CBAM-InvertResNet50 model to detect the content of pork from different parts in adulterated mutton, the models of different pork contents from the back, front leg, and hind leg mixed into mutton were established. The results are shown in Table 1.

It can be obtained from Table 1 that all three models used the CBAM-InvertResNet50 to predict the content of pork from the back, front leg, and hind leg in adulterated mutton have good effects. The values of *R*^2^ were all greater than 0.88, and the RMSE values were all less than 0.38 g·g^−1^. Among them, the effect of mutton mixed with the back dataset was the best, followed by the hind leg dataset, and the prediction effect of the front leg dataset was the worst. The *R*^2^ of the back dataset was 0.9373, and of front leg dataset was 0.8876, with a difference of 0.0497. The results showed that using the RGB image in combination with the CBAM-Invert-ResNet50 could be able to detect the content of the different parts of pork in adulterated mutton, but pork parts had a great influence on the adulteration detection model. This may be caused by some differences in the color, texture, and other aspects among the different parts of the pork. Previous research results showed that different parts of mutton had certain differences in color, texture, and other aspects [11,19].

#### 3.3.2. The Comparison of the Different Models

To verify the superiority of the improved model, the ResNet50, Invert-ResNet50, and CBAM-ResNet50 networks were used to establish the prediction models of different pork content from the back, front leg, and hind leg in adulterated mutton, and the model results were compared with the CBAM-Invert-ResNet50. In addition, the CBAM-Invert-ResNet50 model was compared with the most popular lightweight network MobileNetV3 to verify its reliability. The validation set results of the five models for predicting the content of pork from the back, front leg, and hind leg in adulterated mutton are shown in Table 2.

As shown in Table 2, compared with the ResNet50 and Invert-ResNet50 network models, the CBAM-ResNet50 and CBAM-Invert-ResNet50 network models have large increases in *R*^2^ and decreases in RMSE for three datasets (back, front leg, and hind leg datasets). In the three datasets, the *R*^2^ value of the CBAM-ResNet50 network model was 0.019, 0.1368, and 0.1125 higher than that of the ResNet50 network model, and the RMSE value was 0.0041 g·g^−1^, 0.0155 g·g^−1^ and 0.0147 g·g^−1^ lower than that of the ResNet50 network model, respectively. The *R*^2^ values of the CBAM-Invert-ResNet50 network model for the back dataset, the front leg dataset, and the hind leg dataset were 0.0378, 0.1247, and 0.0391 higher than those of the Invert-ResNet50 network model, respectively. The RMSE values of the CBAM-Invert-ResNet50 network model were 0.0065 g·g^−1^, 0.0125 g·g^−1,^ and 0.0089 g·g^−1^ lower than those of the Invert-ResNet50 network model, respectively. Compared with the CBAM-ResNet50, the *R*^2^ values of the CBAM-Invert-ResNet50 network model for the back and front leg datasets increased by 0.0257 and 0.0102, respectively, and the RMSE values decreased by 0.0033 g·g^−1^ and 0.0010 g·g^−1^, respectively. But the results were slightly lower than the results of the CBAM-ResNet50 for the back dataset. The results showed that adding the attention mechanism CBAM to the ResNet50 and Invert-ResNet50 models could improve the model performance. Our research results were similar to those of Zhang et al. [29] who added the CBAM in the YOLOv4 model to enhance the feature extraction ability of the model, and the results showed that the mAP@0.5 of When they identified the sheep, group1 and group2 were 91.58% and 90.61%, respectively. This was also proved by the study of Du et al. [28]. They incorporated the CBAM in the EfficientNet-B7 model to classify the plug seedling quality. The result showed that the achieved average accuracy of the test set for the proposed model was higher by 7.32% than the accuracy before the improvement. Based on the results in Section 3.2 that the inverted residual structure could make the model meet the requirements of the lightweight, the performance of the improved CBAM-Invert-ResNet50 model was ideal. In addition, compared with MobileNetV3, the *R*^2^ values of the CBAM-Invert-ResNet50 network model for the back and front leg datasets increased by 0.0879, 0.0755, and 0.1657, respectively, and the RMSE values were reduced by 0.0132 g·g^−1^, 0.0087 g·g^−1^ and 0.0181 g·g^−1^, respectively. The results indicated that the improved CBAM-Invert-ResNet50 model was reliable for predicting the content of pork from the back, front leg, and hind leg in adulterated mutton.

#### 3.3.3. Stability Evaluation of the Models

**Figure 8:**
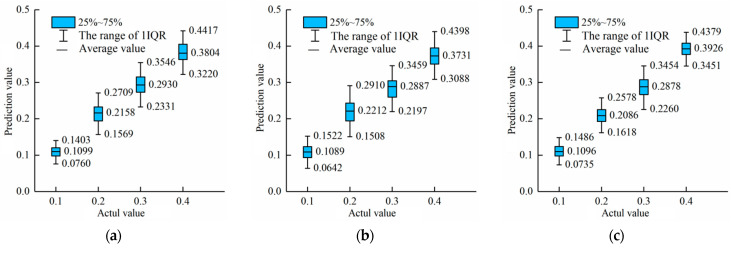

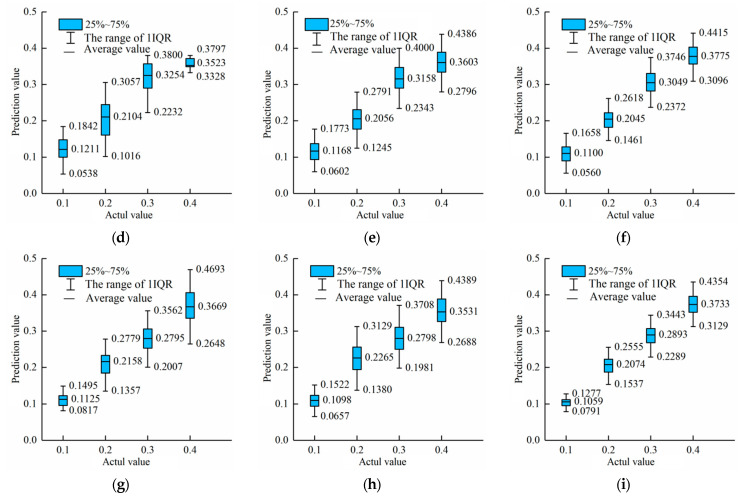
Boxplots of three network models of the MobileNetV3, ResNet50, and CBAM-Invert-ResNet50 for the back, foreleg, and hind leg datasets in the validation set: (**a**) ResNet50 for the back dataset; (**b**) MobileNetV3 for the back dataset; (**c**) CBAM-Invert-ResNet50 for the back dataset; (**d**) ResNet50 for the front leg dataset; (**e**) MobileNetV3 for the front leg dataset; (**f**) CBAM-InvertResNet50 for the front leg dataset; (**g**) ResNet50 for the hind leg dataset; (**h**) MobileNetV3 for the hind leg dataset; (**i**) CBAM-Invert-ResNet50 for the hind leg dataset.

Boxplots were used to visually evaluate the performance of each model in predicting the content of pork from different parts in adulterated mutton. Figure 8 shows the boxplots of the adulterated content prediction values of three models (CBAM-Invert-ResNet50, ResNet50, and MobileNetV3) for the three datasets (back, front leg, and hind leg) of pork-adulterated mutton, respectively.

Figure 8 shows that the predictive values of the CBAM-Invert-ResNet50, ResNet50, and MobileNetV3 are relatively concentrated in the back dataset, and the differences in boxplots among the three are small. Among them, the CBAM-Invert-ResNet50 box is more concentrated than the other two. The boxplots of CBAM-Invert-ResNet50 and MobileNetV3 show little difference in the front leg dataset. In the boxplots of MobileNetV3, the IQR of the predicted value with an adulteration content of 0.4 is small and the data are relatively concentrated. However, when the adulteration content is 20% and 30%, the IQR of the predicted value is too large and the data are scattered. For the hind leg dataset, when the adulterant content is 10%, the IQR of the predicted values for the three models is small, which proves that the three models have a better prediction effect on the hind leg dataset. Among them, the IQR of the CBAM-Invert-ResNet50 is the smallest, which proves that the CBAM-Invert-ResNet50 has the best prediction effect. In addition, for the adulteration content of 20%, 30%, and 40%, the CBAM-Invert-ResNet50 obviously performed better compared with the results of the MobileNetV3 and ResNet50 network models. The above results show that the CBAM-Invert-ResNet50 model had the best stability and significantly better prediction results than ResNet50 and MobileNetV3 in the back dataset, front leg dataset, and back leg dataset.

### 3.4. The Content Detection Model of Mutton Adulterated with Pork from Mixed Parts

#### 3.4.1. Results of the Different Models

**Figure 9:**
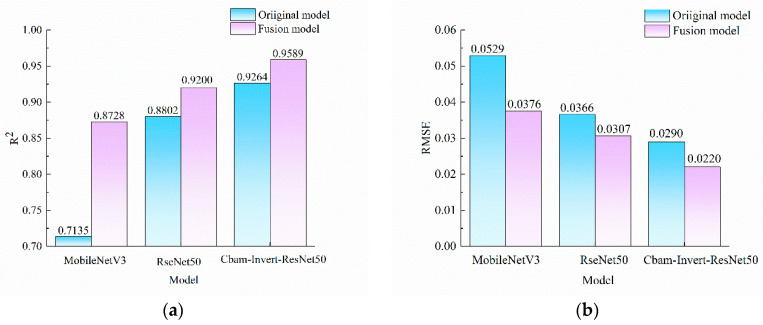
The validation set results of the three models before and after feature fusion for the mixed parts dataset: (**a**) results of *R*^2^; (**b**) results of RMSE.

Table 2 shows that the CBAM-Invert-ResNet50 model had obvious differences in model performance when detecting the content of pork from different parts in adulterated mutton. In order to use the CBAM-Invert-ResNet50 model to accurately detect the content of pork in adulterated mutton in the mixed -dataset, the features of three models, including back, front leg, and back leg, were stitched to eliminate the influence of different parts on the model. To further improve the prediction performance of the model on the mixed-part dataset, transfer learning was used to optimize the pretrained model. After the fusion features were input into the pretrained model, the differences between the fusion features and the actual features were eliminated by fine-tuning. This would ensure that the real features of the mixed-part dataset were further extracted on the basis of making full use of the fused features to improve the accuracy and robustness of the model. At the same time, ResNet50 and MobileNetV3 models were used to establish a feature fusion model to detect the adulteration content in the mixed-part dataset, and the results were compared with those of the CBAM-Invert-ResNet50. The *R*^2^ and RMSE results of the validation set of the three models for the mixed-part dataset before and after feature fusion are shown in Figure 9.

According to Figure 9, before feature fusion, the *R*^2^ values of MobileNetV3, ResNet50, and CBAM-Invert-ResNet50 models for the mixed-part dataset were 0.7133, 0.8802, and 0.9264, respectively. Based on feature fusion, the *R*^2^ values of the MobileNetV3, ResNet50, and CBAM-Invert-ResNet50 combined with transfer learning for the mixed-part dataset were 0.8728, 0.9200, and 0.9589, respectively, with an increase of 0.1595, 0.0398, and 0.0325, respectively, compared with those before feature fusion. The RMSE values of the MobileNetV3, ResNet50, and CBAM-Invert-ResNet50 combined with transfer learning for the mixed-part dataset were reduced by 0.0153 g·g^−1^, 0.0059 g·g^−1^, and 0.0070 g·g^−1^, respectively, compared with those before feature fusion. The above results show that the prediction performance of MobileNetV3, ResNet50, and CBAM-Invert-ResNet50 models based on feature fusion combined with transfer learning was improved on the mixed-part dataset. Among them, the CBAM-Invert-ResNet50 had the best prediction effect on the mixed-part dataset, with *R*^2^ and RMSE of 0.9589 and 0.0220 g·g^−1^, respectively.

#### 3.4.2. Stability Evaluation of the Models

**Figure 10:**
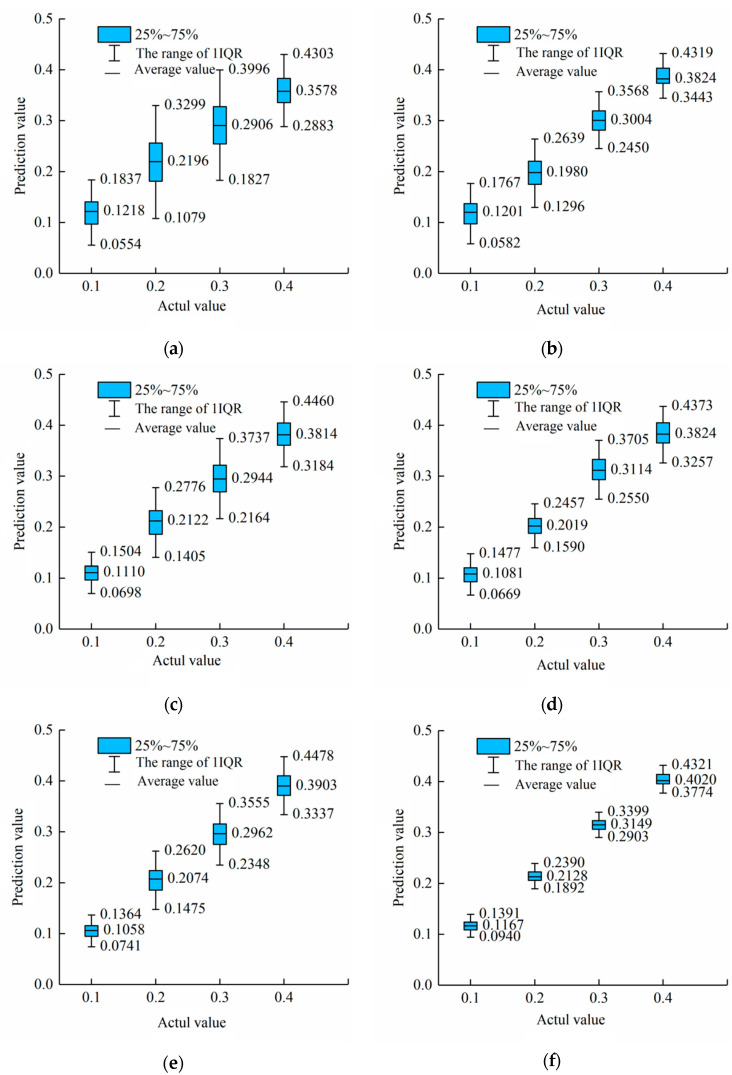
Comparison of boxplots for three network models with mixed-part datasets in the validation set: (**a**) MobileNetV3 model; (**b**) MobileNetV3 model based on feature fusion; (**c**) ResNet50 model; (**d**) ResNet50 model based on feature fusion; (**e**) CBAM-Invert-ResNet50 model; (**f**) CBAM-Invert-ResNet50 model based on feature fusion.

Figure 10 shows the boxplots of the adulterated content predicted by CBAM-Invert-ResNet50, ResNet50, and MobileNetV3 models combined with transfer learning for the mixed dataset before and after feature fusion.

It can be obtained from Figure 10 that the IQR range of the MobileNetV3 model was 0.1079–0.3299 and 0.1827–0.3996, respectively, when the adulteration content was 20% and 30%. The range of the IQR was too large and the data were scattered. The IQR of the MobileNetV3 model based on feature fusion combined with transfer learning for the prediction of 20% and 30% adulterated mutton was significantly reduced and was 0.1296–0.2639 and 0.2450–0.3568, respectively. Similar results were obtained by the ResNet50 model. The IQR of the ResNet50 model based on feature fusion combined with transfer learning for the prediction of 20% and 30% adulterated mutton was significantly reduced, and the range was 0.1590–0.2457 and 0.2550–0.3705, respectively. Compared with the results before the feature fusion, the IQR range of the CBAM-Invert-ResNet50 model based on feature fusion combined with transfer learning for 10%, 20%, 30%, and 40% adulterated mutton was significantly reduced and was 0.0940–0.1391, 0.1892–0.2390, 0.2903–0.3399, and 0.3774–0.4321, respectively. The above results show that the three models, the MobileNetV3, ResNet50, and CBAM-Invert-ResNet50, based on feature fusion combined with transfer learning could improve the stability of the prediction value of the mixed-part dataset. The predicted values were all concentrated. Among them, the CBAM-Invert-ResNet50 had the best prediction stability for the mixed-part dataset.

## 4. Conclusions

The improved CBAM-Invert-ResNet50 model based on inverted residual structure and attention mechanism was used to detect the content of pork from the back, front leg, and hind leg in adulterated mutton under the effect of mutton flavor essence and colorant. Feature fusion and transfer learning were combined to accurately detect the content of pork from mixed parts in adulterated mutton. The results showed that the *R*^2^ of the CBAM-Invert-ResNet50 model for predicting the contents of pork from the back, front leg, and hind leg in adulterated mutton was 0.9373, 0.8876, and 0.9055, respectively, and the RMSE was 0.0268 g·g^−1^, 0.0357 g·g^−1^ and 0.0316 g·g^−1^, respectively. After obtaining the fusion features of different parts by feature stitching, the CBAM-Invert-ResNet50 combined with transfer learning was used to predict the content of pork from mixed parts in adulterated mutton. The *R*^2^ and RMSE were 0.9589 and 0.0220 g·g^−1^, respectively. Compared with that before feature fusion, the *R*^2^ of the mixed-part dataset increased by 0.0325 g·g^−1^ and RMSE decreased by 0.0070 g·g^−1^, respectively. The results showed that the improved CBAM-Invert-ResNet50 model combined with RGB images from mobile phones can be used to quickly and accurately detect the content of pork from specific and mixed parts in adulterated mutton. Among them, the CBAM could effectively increase the feature differences between different content data and significantly improve the accuracy of the prediction model of mutton adulteration content under the effect of additives. Using an inversion residual structure to replace the original residual in the ResNet50 network can make the model more lightweight. For the mixed-part dataset with more complex data features, the feature fusion method could comprehensively utilize multiple image features and complement the advantages of multiple features. Combined with transfer learning, more robust and accurate results could be obtained to predict the content of pork from mixed parts in adulterated mutton. The results of this study can provide guidance for the safety of mutton and its products. At the same time, it promotes the development and application of deep learning combined with image data in the quantitative detection of components of agricultural and livestock products.

## Figures and Tables

**Table 1:** The results of the model for the content of pork from back, front leg, and hind leg adulterated in mutton.

Part	Evaluation Index	Train Set	Test Set	Validation Set
Back	*R* ^2^	0.9609	0.9398	0.9373
	RMSE/g·g^−1^	0.0203	0.0265	0.0268
Front leg	*R* ^2^	0.9699	0.9054	0.8876
	RMSE/g·g^−1^	0.0180	0.0323	0.0378
Hind leg	*R* ^2^	0.9440	0.9119	0.9055
	RMSE/g·g^−1^	0.0244	0.0259	0.0316

**Table 2:** Comparisons of the different models with three datasets of the back, front leg, and hind leg in the validation set.

Models	Back Dataset		Front Leg Dataset		Hind Leg Dataset	
	*R* ^2^	RMSE/g·g^−1^	*R* ^2^	RMSE/g·g^−1^	*R* ^2^	RMSE/g·g^−1^
ResNet50	0.8926	0.0342	0.7406	0.0502	0.7959	0.0457
Invert-ResNet50	0.8995	0.0333	0.7629	0.0482	0.8664	0.0405
CBAM-ResNet50	0.9116	0.0301	0.8774	0.0347	0.9084	0.0317
CBAM-Invert-ResNet50	0.9373	0.0268	0.8876	0.0357	0.9055	0.0316
MobileNetV3	0.8494	0.0400	0.8121	0.0444	0.7398	0.0497

## Data Availability

The data presented in this study are available upon request from the corresponding author.
